# An Eulerian formulation for the computational modeling of phase‐contrast MRI

**DOI:** 10.1002/mrm.30302

**Published:** 2024-09-13

**Authors:** Tomohiro Otani, Tetsuro Sekine, Yu Sato, Ellen Cavalcante Alves, Shigeo Wada

**Affiliations:** ^1^ Department of Mechanical Science and Bioengineering Osaka University Graduate School of Engineering Science Osaka Japan; ^2^ Department of Radiology Nippon Medical School Musashi‐Kosugi Hospital Kanagawa Japan

**Keywords:** Eulerian formulation, high‐performance computing, modified Bloch equation, phase‐contrast MRI

## Abstract

**Purpose:**

Computational simulation of phase‐contrast MRI (PC‐MRI) is an attractive way to physically interpret properties and errors in MRI‐reconstructed flow velocity fields. Recent studies have developed PC‐MRI simulators that solve the Bloch equation, with the magnetization transport being modeled using a Lagrangian approach. Because this method expresses the magnetization as spatial distribution of particles, influences of particle densities and their spatial uniformities on numerical accuracy are well known. This study developed an alternative method for PC‐MRI modeling using an Eulerian approach in which the magnetization is expressed as a spatially smooth continuous function.

**Methods:**

The magnetization motion was described using the Bloch equation with an advection term and computed on a fixed grid using a finite difference method, and k‐space sampling was implemented using the spoiled gradient echo sequence. PC‐MRI scans of a fully developed flow in straight and stenosed cylinders were acquired to provide numerical examples.

**Results:**

Reconstructed flow in a straight cylinder showed excellent agreement with input velocity profiles and mean errors were less than 0.5% of the maximum velocity. Numerical cases of flow in a stenosed cylinder successfully demonstrated the velocity profiles, with displacement artifacts being dependent on scan parameters and intravoxel dephasing due to flow disturbances. These results were in good agreement with those obtained using the Lagrangian approach with a sufficient particle density.

**Conclusion:**

The feasibility of the Eulerian approach to PC‐MRI modeling was successfully demonstrated.

## 1 INTRODUCTION

Phase‐contrast MRI (PC‐MRI) is a powerful tool that provides multidimensional flow velocity fields in living bodies. Nevertheless, it is well known that the MRI‐reconstructed flow velocity field has limited spatiotemporal resolution and is affected by several intrinsic artifacts from multiple sources.^1^, ^2^ Therefore, reasonable interpretation of the reconstructed flow velocity field and its quantitative accuracy remain open problems. Quantitative evaluation of the velocity fields reconstructed using PC‐MRI is challenging because the actual fields are typically unknown, especially for turbulent flows. To address the above concerns, computational simulation of PC‐MRI based on MR physics using prescribed flow velocity fields may provide valuable insights into the physical interpretation of MRI‐reconstructed velocity fields and underlying origins of measurement errors.

Computational modeling of conventional MRI is well established^3^, ^4^, ^5^, ^6^ and involves determining the motion of the magnetization vector (i.e., the spin isochromat), described using the Bloch equation,^7^ for a given pulse sequence. In contrast, computational modeling of PC‐MRI remains limited because of the complexities in appropriately treating the magnetization transport caused by background advection.^8^ There are two main computational approaches to treating the magnetization transport: Lagrangian and Eulerian. Almost all recent studies attempting the computational simulation of PC‐MRI adopted the Lagrangian approach,^9^, ^10^, ^11^, ^12^, ^13^ as summarized by Puiseux et al.^13^ In the Lagrangian approach, the magnetization is represented as a spatial distribution of particles whose trajectories are computed in the prescribed flow velocity fields. Because the magnetization motion can be expressed using the original Bloch equation by tracking the trajectory of each particle, the Lagrangian approach can be intuitively and easily implemented by extending a conventional MRI simulator.^12^ One drawback of this approach is that low particle densities and spatial nonuniformities strongly affect the PC‐MRI simulation outputs,^12^, ^13^ and this drawback may be critical in the PC‐MRI simulation of complex velocity fields.

The alternative Eulerian approach expresses the magnetization as a smooth, continuous function in space and time and computes the magnetization transport on a fixed grid (Eulerian coordinates). This method requires a modification of the Bloch equation to incorporate the advection effect and a suitable computational scheme to solve the modified partial differential equation. One advantage of this approach is that the PC‐MRI simulation can be performed free from particle inhomogeneity effects that limit in Lagrangian approach.^10^, ^12^, ^13^ However, the Eulerian approach has been used in only a few basic computational studies of PC‐MRI^14^, ^15^ and MR angiography.^16^ Thus, the feasibility of the Eulerian approach to computational modeling of PC‐MRI, especially for complex flow fields, has not been well demonstrated, as pointed out elsewhere.^10^, ^12^, ^13^ The establishment of efficient computational models of PC‐MRI using the Eulerian approach may provide a valuable alternative to address the drawbacks of current PC‐MRI models.

This study aimed to develop a computational model of PC‐MRI using the Eulerian approach and demonstrate its feasibility. An efficient formulation for solving the modified Bloch equation on a fixed grid was derived using reasonable assumptions. Computational simulations of PC‐MRI experiments using both Eulerian and Lagrangian approaches were conducted and compared using three numerical examples with prescribed velocity fields obtained from analytical and computational solutions of the incompressible fluid flow equations.

## 2 METHODS

Computational simulations of PC‐MRI used the spoiled gradient echo (SPGR) sequence,^2^ and the velocity field was reconstructed using a simple four‐point method.^17^ Section 2.1 derives the general formulation of the PC‐MRI model, including the magnetization motion during the imaging sequence and signal sampling, and image reconstruction. Section 2.2 describes the computational implementation using Lagrangian and Eulerian approaches. The Eulerian formulation partly follows the existing model of magnetic resonance angiography,^16^ with modifications made for PC‐MRI, and the Lagrangian formulation is based on the approach of ref.^13^ Section 2.3 introduces numerical examples of a fully developed cylindrical flow^13^ and flow in a cylinder with stenosis.^18^

### 2.1 Formulations

A computational domain Ω is defined in a Cartesian coordinate system (x,y,z) with the static magnetic field directed along the z‐axis. Assuming the magnetization vector $M={\left(M_{x},M_{y},M_{z}\right)}^{⊤}$ is set in a prescribed flow velocity field of an incompressible fluid, the Bloch equation in the rotating frame of reference is given by

(1) $$\frac{DM}{Dt}=\gamma M\times B-\frac{M_{x}}{T_{2}}e_{x}-\frac{M_{y}}{T_{2}}e_{y}+\frac{M_{0}-M_{z}}{T_{1}}e_{z},$$

where γ is the gyromagnetic ratio, B is the external magnetic field vector, $T_{1}$ and $T_{2}$ are the respective longitudinal and transverse relaxation times, $M_{0}$ is the magnitude of the steady‐state magnetization, $e_{x}$, $e_{y}$, and $e_{z}$ are unit orthogonal vectors, and D/Dt is the material derivative, given by

(2) $$\frac{D}{Dt}:=\frac{\partial }{\partial t}+v\cdot \nabla ,$$

where v is the background flow velocity vector set as continuos function in space and time. The magnetic field B is divided into three terms:

(3) $$B=B_{x}e_{x}+B_{y}e_{y}+G\cdot xe_{z},$$

where $B_{x}$ and $B_{y}$ are the radiofrequency (RF) magnetic field components in the transverse plane, G is the linear magnetic field gradient, and x is the position vector.

For subsequent formulation, transverse components $M_{x}$ and $M_{y}$ are rewritten in cylindrical coordinates (r,ϕ):

(4) $$M_{x}=M_{r}\cos\phi ,M_{y}=M_{r}\sin\phi ,$$

where $M_{r}$ is the magnitude of transverse magnetization and ϕ is the associated phase along the z‐axis.

#### 2.1.1 Excitation

Magnetic excitation induced by an RF pulse was modeled as follows. Because the duration τ of the exciting RF pulse is sufficiently short on the timescales of both relaxation and flow transport, the excitation step was expressed as a pure rotation of M, given by

(5) $$M^{+}=R_{\varphi }(\alpha )M^{-},$$

where the superscripts − and + denote the times before and after the RF pulse, respectively, and R(α) is the rotation matrix corresponding to the flip angle $\alpha =\gamma B_{r}\tau$ around the axis with an azimuthal angle φ in the transverse plane.

Furthermore, we assumed that $M_{r}$ and ϕ were zero before each repetition of the SPGR sequence. Thus, Equation (6) can be further simplified as

(6) $$M_{z}^{+}=M_{z}^{-}\cos\alpha ,M_{r}^{+}=M_{z}^{-}\sin\alpha ,\phi^{+}=0.$$

#### 2.1.2 Relaxation

In the relaxation step, only the z‐component of B is non‐zero value during the spatial/velocity‐encoding period following the RF pulse: $B={\left(0,0,B_{z}\right)}^{⊤}$, where $B_{z}=G\cdot x$ from Equation (3). Thus, Equation (1) can be split into three independent scalar advection equations:

(7) $$\begin{array}{ll} \frac{DM_{z}}{Dt} & =\frac{M_{0}-M_{z}}{T_{1}}, \end{array}$$

(8) $$\begin{array}{ll} \frac{DM_{r}}{Dt} & =-\frac{M_{r}}{T_{2}}, \end{array}$$

(9) $$\begin{array}{ll} \frac{D\phi }{Dt} & =-\gamma B_{z}. \end{array}$$

**FIGURE 1:**
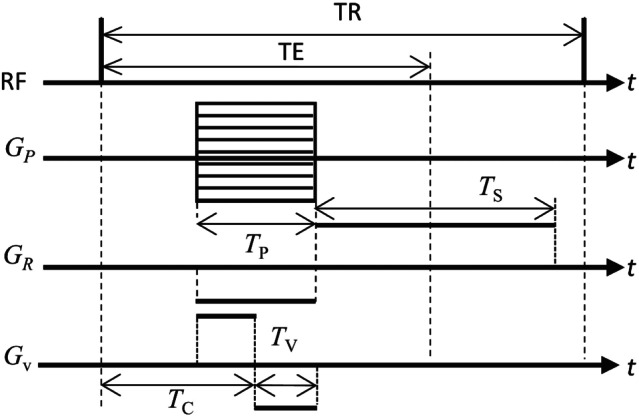
Diagram of phase‐encoding ($G_{P}$), frequency‐encoding ($G_{R}$), and velocity‐encoding ($G_{V}$) gradients in the spoiled gradient echo sequence, which have durations of $T_{C},T_{P},T_{S}$, and $T_{V}$, respectively. TR, repetition time; TE, echo time; RF, radiofrequency.

For the magnetic field gradient G, we assigned the frequency‐encoding gradient, $G_{R}$, to the x component and the phase‐encoding gradient, $G_{P}$, to the other independent components. A bipolar gradient (BPG) for velocity encoding, $G_{V}$, was applied in each direction following a simple four‐point method. Figure 1 shows a diagram of the magnetic field gradients in the SPGR sequence. In a two‐dimensional case in the xy plane, $G_{R}$, the maximum value of $G_{P}$ ($G_{P}^{\max}$), and $G_{V}$ are^2^

(10) $$G_{R}=\frac{2\pi }{\gamma L_{x}T_{s}},G_{P}^{\max}=\frac{2\pi }{\gamma L_{y}T_{P}},G_{V}=\frac{\pi }{\gamma v_{\text{venc}}T_{V}^{2}},$$

where $L_{x}$ and $L_{y}$ show a field of view (FOV), $T_{S},T_{P}$, and $T_{V}$ are the durations of the frequency‐encoding, phase‐encoding, and velocity‐encoding gradients, respectively, and $v_{\text{venc}}$ is the maximum velocity for encoding. Each gradient component was simplified as a rectangular function.

The MRI signal, $S=S_{\text{Re}}+iS_{\text{Im}}$, was collected during $G_{R}$ exposure by computing the volume integrals of the transverse magnetization given by

(11) $$S_{\text{Re}}=\int_{\Omega }M_{r}\cos\phi d\Omega ,S_{\text{Im}}=\int_{\Omega }M_{r}\sin\phi d\Omega .$$

Full k‐space sampling was performed in a sequential order. Image reconstruction from the MRI signal was conducted using an inverse Fourier transform, and the velocity field was computed following a simple four‐point method.^17^

### 2.2 Computations

In both the Lagrangian and Eulerian approaches, the computational domain Ω was spatially discretized on a rectangular Cartesian grid, and the background velocity field v was assigned on the grid. In addition, the magnetic field $B_{z}$ was set as a prescribed function of space and time. The magnetization motion (Equations (7), (8), (9)) was computed step‐by‐step using an explicit fourth‐order Dormand–Prince scheme^19^ until k‐space sampling was completed. The time interval was restricted to maintain a Courant–Friedrichs–Lewy number <1, which is a necessary condition for the stability of an explicit numerical scheme about the spatial and temporal discretization steps (cf. Reference 20). Further details on each approach are noted below.

#### 2.2.1 Lagrangian approach

In the Lagrangian approach, M is expressed as a set of massless particles with magnetization $M(x^{p};t)$, (p=1,...,N) randomly distributed in Ω in the initial state. With this definition, Equations (7)–(9) can be directly solved as a set of ordinary differential equations to obtain the position vector of each particle:

(12) $${\left.\frac{dx^{p}}{dt}\right|}_{t}=v(x^{p},t).$$

Here, the particle velocity was determined by linear interpolation of the background velocity field at each time step. In addition, the MRI signal in Equation (11) was converted from a volume integral to a summation over all particles:

(13) $$S_{\text{Re}}=\overset{N}{\underset{p=1}{\sum }}M_{r}^{p}\cos\phi^{p}\Delta V^{p},S_{\text{Im}}=\overset{N}{\underset{p=1}{\sum }}M_{r}^{p}\sin\phi^{p}\Delta V^{p},$$

where ΔV is the particle volume determined from particle density in the initial state.

For computational efficiency, we did not add particles entering from inlets or remove particles exiting through outlets during the computation of the MRI scan. Instead, we ensured that the computational domain provided a sufficient margin around the FOV on both the upstream and downstream sides.

#### 2.2.2 Eulerian approach

In the Eulerian approach, M is expressed as a spatiotemporally smooth, continuous function $\left(M:\Omega \times [0,\infty )\to {\mathbb{R}}^{3}\right)$ in fixed coordinates, and the material derivatives in Equations (7)–(9) are expressed in a partial differential form:

(14) $$\begin{array}{ll} \frac{\partial M_{z}}{\partial t}+v\cdot \nabla M_{z} & =\frac{M_{0}-M_{z}}{T_{1}}, \end{array}$$

(15) $$\begin{array}{ll} \frac{\partial M_{r}}{\partial t}+v\cdot \nabla M_{r} & =-\frac{M_{r}}{T_{2}}, \end{array}$$

(16) $$\begin{array}{ll} \frac{\partial \phi }{\partial t}+v\cdot \nabla \phi & =-\gamma B_{z}. \end{array}$$

Variables $M_{z}$, $M_{r}$, and ϕ were defined at each grid point, and Equations (14)–(16) were discretized using a finite difference method, with the advection term being spatially discretized using a fifth‐order weighted essentially nonoscillatory scheme.^21^, ^22^ As the boundary condition, we imposed $M_{z}=M_{0}$, $M_{r}=0$, and ϕ=0 on the inlet boundaries and ∂M/∂n=0 on the outlet boundaries, where n is the unit outer normal. The MRI signal in Equation (11) was computed as a summation over the volume integrals of all grids $\Omega^{e}$, given by

(17) $$S_{\text{Re}}=\underset{e}{\sum }\int_{\Omega^{e}}M_{r}\cos\phi d\Omega ,S_{\text{Im}}=\underset{e}{\sum }\int_{\Omega^{e}}M_{r}\sin\phi d\Omega .$$

Here, M was linearly interpolated on each grid.

### 2.3 Numerical examples

Three numerical examples of the PC‐MRI computations were performed. In all examples, we set γ = 42.58 MHz/T, $T_{1}$ = 1200 ms, and $T_{2}$ = 250 ms in Ω. Unless otherwise stated, the following parameters were used for the spatial/velocity encoding: $T_{S}=N_{x}\Delta t_{s}$, where $N_{x}$ is the number of voxels in the x‐direction and $\Delta t_{s}$ = 0.01 ms is the sampling pitch; $T_{P}=0.5T_{S}$; and $T_{V}=0.5T_{P}$. The BPG was applied during the phase encoding period, as shown in Figure 1. For simplicity, M was normalized such that $M_{0}$ = 100, and magnetic field inhomogeneity was neglected. In addition, we assumed that magnetization in Ω was saturated in the initial state ($M_{z}=M_{s}$), where

(18) $$M_{s}=M_{0}\frac{1-\exp(-\text{TR}/T_{1})}{1-\cos\alpha \exp(-\text{TR}/T_{1})}.$$

Here, TR is the repetition time (Figure 1).

#### 2.3.1 Hagen–Poiseuille flow

**FIGURE 2:**
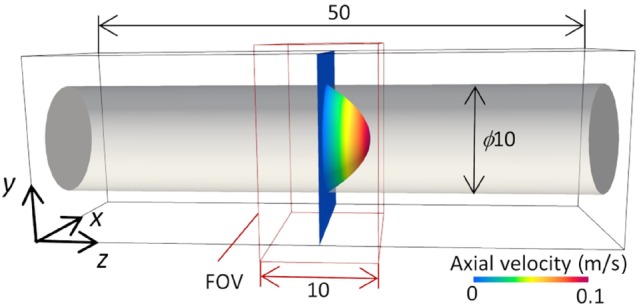
Fully developed cylindrical flow in a computational domain (black outline) encompassing the field of view (FOV) of the MRI scan (red outline).

To verify the Lagrangian and Eulerian approaches to modeling PC‐MRI, we first considered a fully developed flow along the longitudinal (z) axis of a straight cylinder (Hagen–Poiseuille flow), following Reference 13 A cylinder radius $r_{0}$ = 5 cm and axial length of 50 cm was set in a rectangular computational domain of 16 mm × 16 mm × 50 mm (Figure 2). Here, the axial velocity, $v_{z}(r)$, in cylindrical coordinates is given by

(19) $$v_{z}(r)=v_{\max}\left(1-{\left(\frac{r}{r_{0}}\right)}^{2}\right),$$

where $v_{\max}$ is the maximum analytical velocity, which was set to 0.1 m/s. The scanning parameters were: TR, 5 ms; echo time (TE), 4 ms; acquired matrix, 36 × 36; FOV, 18 mm × 18 mm × 10 mm; voxel size, 0.5 mm × 0.5 mm × 10 mm; flip angle, 4° (determined from the Ernst angle^2^); $v_{\text{venc}}$, 0.12 m/s. Because flow velocity along the radial direction was zero, we reconstructed only $v_{z}(r)$ using a two‐point method.

The velocity field reconstructed from the PC‐MRI simulation was compared with the analytical solution (spatially averaged in each voxel) using the following error function^13^:

(20) $$\text{error}=\left|\frac{v_{\text{MRI}}-v_{z}}{v_{\max}}\right|,$$

where $v_{\text{MRI}}$ is the MRI‐reconstructed axial velocity. This error was computed in each voxel, and mean and maximum errors in MRI images were obtained.

The sensitivity of the output to the grid size was assessed in each approach. In the Eulerian approach, computations were run using three grid densities: a coarse grid equivalent to the MRI voxel size (32 × 32 × 5); a medium grid (64 × 64 × 10); and a fine grid (128 × 128 × 20). In addition, in the Lagrangian approach, computations were run using three particle densities: 1, 10, and 100 particles/voxel. These computations were parallelized using 12 openMP cores on Intel(R) Xeon(R) Silver 421R CPU@2.40 GHz.

#### 2.3.2 Flow in a cylinder with stenosis

Next, we conducted PC‐MRI simulations of the flow velocity field in a straight axisymmetric cylinder with stenosis, a geometry previously used to compare PC‐MRI simulations.^10^ The radius $\hat{r}(z)$ of the stenosis was expressed as

(21) $$\frac{\hat{r}}{{\hat{r}}_{0}}=\left\{\begin{array}{ll} 1-(1+\cos(z\pi /2))/4\; & -L<z<L\\ 1\; & |z|\geq L \end{array}\right.,$$

where L is the diameter of the cylinder (= $2{\hat{r}}_{0}$) as a characteristic length and was set to 14.6 mm. The axial length of the cylinder was set to 2L on the anterior side of the stenosis and 13L on the posterior side.

**FIGURE 3:**
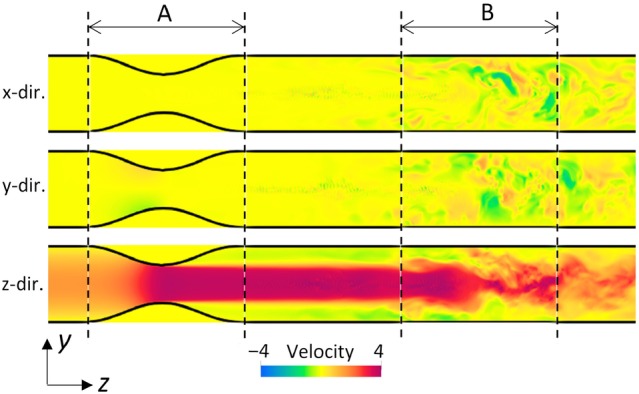
Spatial velocity distribution in a cylinder with stenosis along each direction in the yz midplane, obtained at t=0.5 s after the initial state. Target computational domains are shown in A (stenotic flow domain) and B (disturbed flow domain).

To obtain the prescribed flow velocity field for the PC‐MRI simulations, we conducted a computational fluid dynamics (CFD) simulation using a Cartesian grid solver developed in our previous studies.^23^, ^24^ The computational domain was a 15 mm × 15 mm × 225 mm cuboid and discretized using a 128 × 128 × 1920 Cartesian grid with a uniform step size of approximately 0.12 mm. The flow was modeled as an incompressible Newtonian fluid using the incompressible Navier–Stokes equation with a kinematic viscosity of 0.01 cm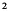/s. This governing equation was updated in time on a conventional staggered grid in a finite difference manner. The flow field, with mechanical interactions between the solid and fluid domains on the Cartesian grid, was calculated using the boundary data immersion method.^25^ For the CFD simulations, we assumed a fully developed cylindrical flow (Equation 19) with a Reynolds number of 1000, 20% turbulent intensity at the inlet, and zero pressure at the outlet. The CFD computation was conducted for 0.5 s, with confirmation that the flow disturbances downstream from the stenosis were fully developed. The time increment was chosen to ensure a Courant–Friedrichs–Lewy number <0.5. This computation was conducted using hybrid parallelization with 12 OpenMP cores and 960 message passing interface (MPI) nodes on the supercomputer Fugaku (RIKEN). The flow velocity field at t=0.5 s after the initial state is illustrated in Figure 3. A sufficient flow developed around the stenosis, and flow disturbances appeared downstream from the stenosis.

As numerical examples, we selected two domains (A and B in Figure 3) as targets for computation. First, MRI scans of the flow in the anterior side of the stenosis (domain A) were simulated under different measurement conditions to assess displacement artifacts (i.e., misregistration artifacts).^26^ Next, MRI scans of the disturbed flow in the posterior side of the stenosis (domain B) were simulated, and the extent of flow disturbance was assessed using the intravoxel velocity SD (IVSD).^18^, ^27^ These computations were conducted using the Eulerian approach to demonstrate its feasibility, and computations using Lagrangian approach were partly conducted for comparison.

Note that we used the instantaneous velocity field at t = 0.5 s after the initial state as the prescribed velocity field because these numerical examples did not aim to achieve actual time‐resolved flow but evaluate the feasibility of the Eulerian approach for PC‐MRI modeling of spatially complex flow fields. Furthermore, positions and states of magnetization were reinitialized to their respective steady‐state values before each repetition of the pulse sequence, following Reference 13.

Computations using the Eulerian approach were conducted using hybrid parallelization with 12 OpenMP cores and 128 MPI nodes on the supercomputer Fugaku. In contrast, computations using the Lagrangian approach were performed using 16 openMP cores on Intel(R) Xeon(R) Gold 6326 CPU @ 2.90GHz.

##### Domain A: displacement artifact

Two‐dimensional MRI scans of the flow on the anterior side of the stenosis (domain A in Figure 3) were performed to assess the effects of displacement artifacts. Following References 26 and 28, the MRI reconstructed velocities with displacement artifacts is estimated through streamline of each magnetization in Lagrangian form. In one‐dimensional case, the MRI reconstructed velocity $v_{\text{MRI}}$ can be interpreted to be $v(T_{C})$, where $T_{C}$ is the length of time between the end of the RF pulse and the center of the BPG (Figure 1). Following this idea, $v_{\text{MRI}}$ of magnetization $x^{p}$ at t= TE in the three‐dimensional case can be estimated as

(22) $$v_{\text{MRI}}(x^{p}(\text{TE}))=v(x^{p}(T_{C})).$$

Thus, the extent of displacement artifacts can be estimated from $T_{C}$ and TE.

MRI simulations using the Eulerian approach were conducted using the following scanning parameters: acquired matrix, 32 × 32; FOV, 15 mm × 15 mm × 2 mm; voxel size, 0.4685 mm × 0.4685 mm × 2 mm; flip angle, 5°; $v_{\text{venc}}$, 4 m/s. The computational grid was the same as that used for the CFD simulation in domain A (128×128×256). To assess the displacement artifacts in the MRI‐reconstructed velocity field, we simulated four cases, with TE 1, 2, 3, and 4 ms.

For comparison, MRI simulations were also conducted using the Lagrangian approach under the same computational conditions described above, together with TE = 4 ms and 10, 100, and 1000 particles/voxel.

##### Domain B: IVSD

Two‐dimensional MRI scans of the flow in the region on the posterior side of the stenosis (domain B in Figure 3) were performed to assess the extent of flow disturbances using the IVSD of the flow in the x and y directions perpendicular to the main flow. The IVSD was computed assuming that the distribution of flow velocities in each voxel was Gaussian^18^, ^27^ in each direction and given by

(23) $$\text{IVSD}=\sqrt{\frac{2\ln\left(\frac{\left\|I_{0}\right\|}{\left\|I_{\text{BPG}}\right\|}\right)}{{\left(\pi v_{\text{venc}}\right)}^{2}}},$$

where $I_{\text{BPG}}$ and $I_{0}$ are the reconstructed MRI signals with and without the BPG, respectively.

We used a short TE of 0.5 ms to limit the size of displacement artifacts. Other scanning parameters were: acquired matrix, 10 × 10; voxel size, 2 mm × 2 mm × 2 mm; flip angle, 5°; $v_{\text{venc}}$, 1.5 m/s. In the Eulerian approach, the computational grid was the same as that used for the CFD simulation of domain B (128×128×256). For comparison, MRI simulations were also conducted using the Lagrangian approach with 1000 particles/voxel.

## 3 RESULTS

### 3.1 Hagen–Poiseuille flow

**FIGURE 4:**
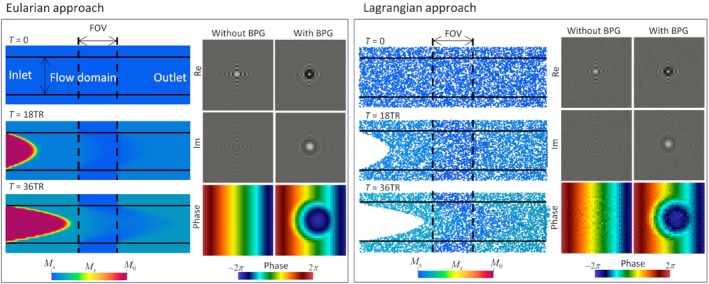
Workflow for phase‐contrast MRI simulation of fully developed cylindrical flow using the Eulerian approach (left) and Lagrangian approach (right). The magnetization was assumed to be saturated ($M_{z}=M_{s}$) at the initial state (t=0) and transported at the background flow velocity during scanning with excitation only in the field of view. Shown are the k‐space and resulting phase images in cases with and without applied bipolar gradients. The Lagrangian images show a representative example with 10 particles/voxel.

Figure 4 summarizes the workflow of the PC‐MRI simulations of the fully developed cylindrical flow using the Eulerian approach (left) and Lagrangian approach (right). The motion of the magnetization excited by the RF pulse was computed in the presence of an external magnetic field and flow advection. The k‐space sampling was performed with and without BPGs, and then each phase image was reconstructed. Although the Lagrangian approach in this study did not consider magnetization transport from the inlet, the Eulerian approach showed that such transport occurs for the unsaturated magnetization $M_{z}=M_{0}$.

**FIGURE 5:**
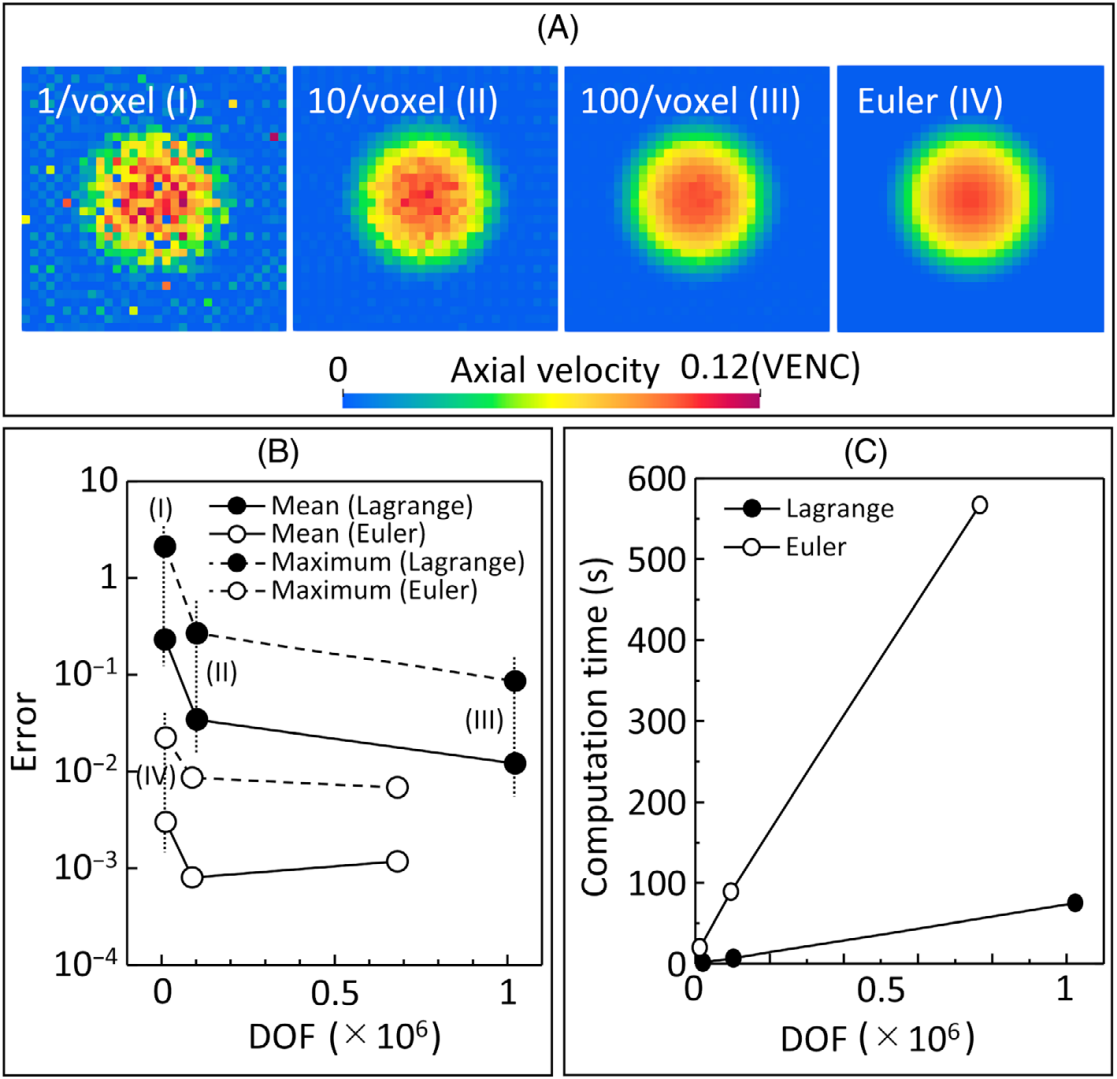
Comparisons of the MRI reconstructed velocity fields using Eulerian and Lagrangian approaches. (A) Reconstructed velocity fields obtained using the Lagrangian approach with 1 (I), 10 (II), and 100 (III) particles/voxel and using the Eulerian approach with a coarse grid (32 × 32 × 5, (IV)). (B) Maximum and mean errors obtained using different degrees of freedom (DOF) in the Lagrangian and Eulerian approaches. (C) Dependence of the computational time on the DOF.

Figure 5A shows the flow velocity fields reconstructed using the Lagrangian approach with particle densities of 1, 10, and 100 particles/voxel and using the Eulerian approach with a coarse grid. Using the Lagrangian approach, the velocity profiles obtained with a low particle density contained irregular errors, but those obtained with larger particle densities were smoother. In contrast, the velocity fields reconstructed using the Eulerian approach were in excellent agreement with the input velocity fields in all three cases with different grid sizes, and the errors in the center of the cylinder were less than 0.5% even when coarse grids were used. The mean and maximum errors of the reconstructed velocities are shown in Figure 5B for each approach. Using the Lagrangian approach, the maximum and mean errors decreased with an increasing particle density, and the maximum error was less than $10^{-2}$ in the case of 100 particles/voxel. Using the Eulerian approach, both the maximum and mean errors were one order of magnitude lower than those using the Lagrangian approach with similar degrees of freedom (DOF), and the mean error converged to $10^{-4}$. The computational time taken for each MRI simulation is shown in Figure 5C. The computational time increased in proportion to the DOF using both Eulerian and Lagrangian approaches. The rate of the increase in the Eulerian approach was approximately $0.86\times 10^{-3}$ s/DOF and an order of magnitude higher than that for the Lagrangian approach ($0.08\times 10^{-3}$ s/DOF). Whereas, the computational time using the Eulerian approach with a coarse grid was 21 s, which was shorter than that of the Lagrangian approach with particle densities of 100 particles/voxel (82 s) despite relatively low DOF and computational errors.

### 3.2 Flow in a cylinder with stenosis

#### 3.2.1 Displacement artifact

**FIGURE 6:**
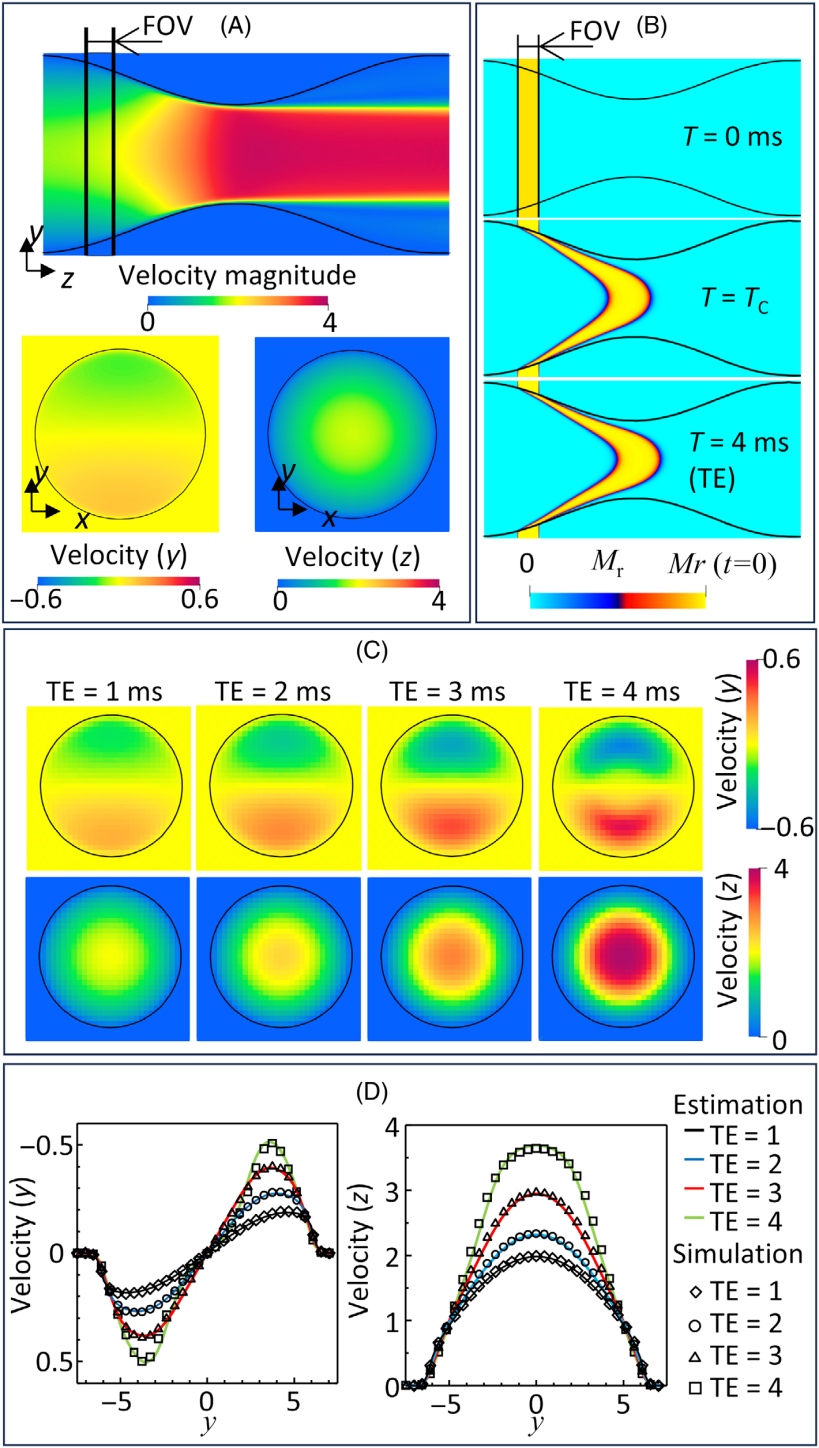
Summary of the displacement artifacts in the MRI‐reconstructed flow velocity fields obtained using Eulerian approach. (A) Input velocity distributions at the center of the field of view (FOV) in the cross‐sectional area of target flow domain (domain A in Figure 3). (B) Representative snapshots of the spatial distribution of the transverse magnetization at t=0, $T_{C}$, and echo time (TE). (C) Reconstructed velocity fields obtained using TE of 1, 2, 3, and 4 ms. (D) Estimated and reconstructed cross‐sectional velocity profiles at the center of the FOV.

Figure 6 summarizes MRI simulations of stenosed flow using the Eulerian approach. The FOV was set on the anterior side of the stenosis (Figure 6A). Since the flow was accelerated and had a radial velocity distribution through the stenosis, the excited magnetization was concentrated in the center of the cylinder during the pulse sequence (Figure 6B). Figure 6C shows that the reconstructed velocity fields increased with increasing TE. Velocity profiles at the center of the FOV are plotted along the y‐axis in Figure 6D, which shows that the estimated and reconstructed profiles were in excellent agreement regardless of TE.

**FIGURE 7:**
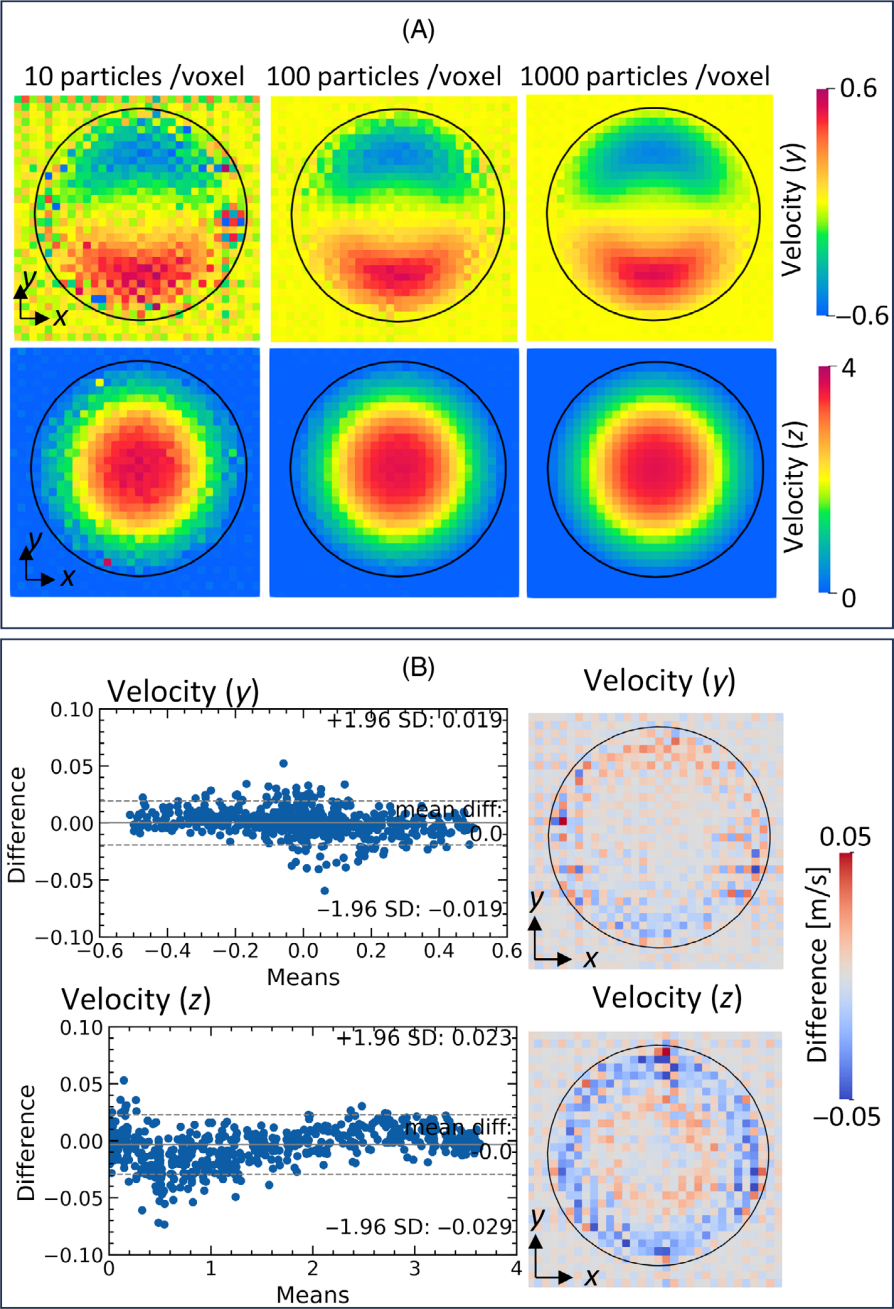
Comparison of the MRI‐reconstructed flow velocity profiles obtained using the Eulerian and Lagrangian approaches. (A) Reconstructed velocity distribution obtained using the Lagrangian approach with 10, 100, and 1000 particles/voxel. (B) Bland–Altman plots of the reconstructed velocity components in the y and z directions, obtained using the Eulerian and Lagrangian approaches (left), and spatial distributions of the differences in each velocity component between these approaches (right).

For comparison, Figure 7A shows the reconstructed velocity fields (TE = 4 ms) using the Lagrangian approach with 10, 100, and 1000 particles/voxel. With 10 particles/voxel, the reconstructed velocities around the center of the stenosis were in good agreement with those obtained using the Eulerian approach, but the velocities around the wall had non‐negligible errors. Although these errors could be limited by using a larger particle density, the velocity field had a slight checkerboard appearance even using 1000 particles/voxel. Figure 7B shows Bland–Altman plots of the reconstructed velocities using the Eulerian approach and Lagrangian approach (1000 particles/voxel) as well as the spatial distributions of the differences between them. Mean values of the differences were close to zero, and the differences were mainly distributed in the ±0.05 m/s range. Relatively high errors centered on the mean values of 0 m/s in both the y and z velocity components were mainly distributed near the wall with a checkerboard profile.

#### 3.2.2 Intravoxel velocity SD

**FIGURE 8:**
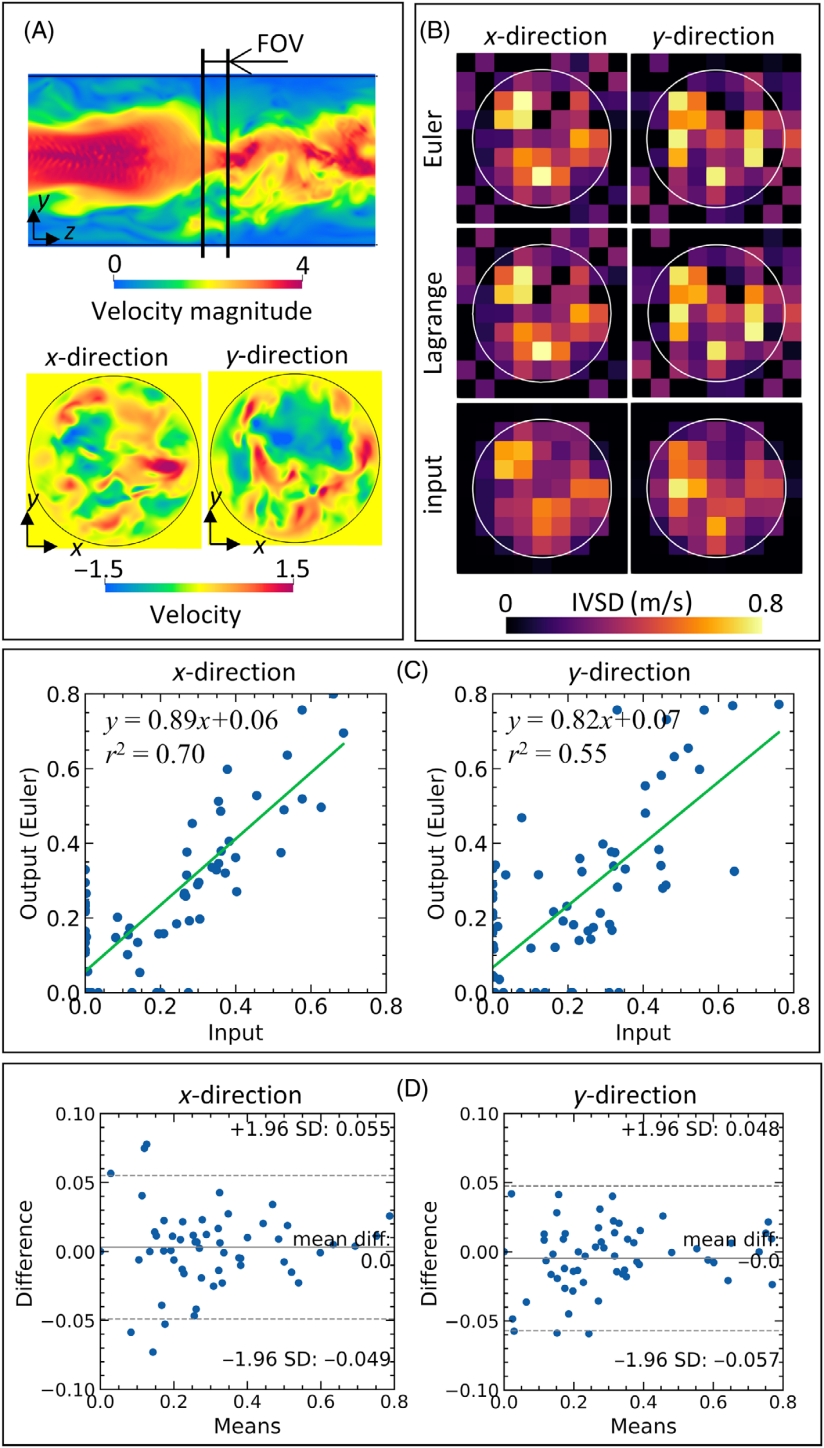
Summary of the intravoxel velocity standard deviation (IVSD) evaluations. (A) Input velocity distributions at the center of the field of view (FOV) in the cross‐sectional area of target flow domain (domain B in Figure 3). (B) Spatial distributions of the IVSD for the x and y components of the input velocity field and the fields reconstructed using the Eulerian and Lagrangian approaches. (C) Linear regression analyses of the correlation between the IVSDs of the input and MRI‐reconstructed (Eulerian approach) fields in each direction. (D) Bland–Altman plots of the differences in the IVSDs between the Eulerian and Lagrangian approaches.

Figure 8 summarizes the IVSD‐based evaluations of the Eulerian and Lagrangian approaches. The FOV was set at the center of the target computational domain (Figure 8A). Figure 8B shows the spatial dependence of the IVSD for the x and y components of the flow velocities obtained from MRI simulations using Eulerian and Lagrangian approaches. As a reference, it also shows the corresponding IVSDs for the flow velocities from the input velocity fields. Relatively large IVSDs values were found at the same locations for both input and MRI‐reconstructed profiles. Correlations between the input velocity field and the MRI‐reconstructed field obtained using the Eulerian approach were assessed using linear regression analyses (Figure 8C). Strong positive relationships were found, with linear regression coefficients of 0.89 (x component) and 0.82 (y component) and $r^{2}$ values of 0.70 (x component) and 0.55 (y component). Figure 8D shows Bland–Altman plots of the IVSD using the Eulerian and Lagrangian approaches. The differences in these values obtained using each approach were mainly distributed in ±0.05 m/s. The mean values of the differences were close to zero, and almost all values were distributed within the confidence interval.

## 4 DISCUSSION

Computational modeling of PC‐MRI is expected to improve our understanding of the MRI‐reconstructed flow velocity field and the physical origins of its measurement errors. Previous PC‐MRI simulations have used a Lagrangian approach to solve the Bloch equation in the prescribed flow field.^9^, ^10^, ^11^, ^12^, ^13^ Nevertheless, drawbacks of this approach have been identified, including quantification of accuracies and Lagrange particle inhomogeneities due to the flow transport.^10^, ^12^, ^13^ Thus, this study investigated the feasibility of an alternative Eulerian approach to the computational modeling of PC‐MRI. An efficient formulation was used to solve the modified Bloch equation as three independent scalar advection equations with a discretization scheme that has been well established in the field of computational mechanics,^29^ and three numerical examples were provided. The results showed that Eulerian approach yielded reasonable flow profiles in all numerical examples and successfully demonstrated its feasibility for PC‐MRI simulation.

In the case of fully developed cylindrical flow, the velocity profiles reconstructed using the Eulerian approach showed excellent agreement with the analytical solution even when using a coarse grid size (Figure 4). In contrast, those obtained using the Lagrangian approach strongly depended on the Lagrange particle density (Figure 5). These findings are consistent with earlier studies using the Lagrangian approach,^12^, ^13^ and the range of errors observed in this study are consistent with those reported by Reference 13 Because the Lagrangian approach models the volume integral used for MRI signal computation as a summation over all Lagrange particles, a sufficient particle density is required (10–1000 particles/voxel^12^, ^13^). In contrast, the Eulerian approach expresses the magnetization as a spatially smooth, continuous function, and thus the volume integral can be computed directly and with sufficient numerical accuracy if the prescribed velocity fields are well resolved on the computational grid. Based on these characteristics, the Eulerian approach may be advantageous when magnetization transport during the MRI pulse sequence is significant, in which case the Lagrange particle inhomogeneity becomes severe.

Following the above perspective, we further evaluated the extent of displacement artifacts, which are commonly observed in MRI of stenosed flow^26^, ^28^, ^30^ and caused by magnetization transport during pulse sequence. The results successfully demonstrated that the velocity fields reconstructed using the Eulerian approach were in excellent agreement with those estimated from theory (Figure 6). Although the Lagrangian approach could also produce consistent flow velocity fields with displacement artifacts, while the resulting velocity fields contained checkerboard profile around the wall even when a density of 1000 particles/voxel was used (Figure 7). As the flow concentrates in the stenosed region, the Lagrangian particle inhomogeneity around the wall may increase and cause these errors. These results highlight the advantage of the Eulerian approach, which is free from such limitations.

The Lagrangian approach has a strong advantage in terms of computational cost, especially in cases where the magnetization motion can be simplified by assuming it is reinitialized before each repetition of the pulse sequence and in cases where limited magnetization transport occurs during the pulse sequence. In these situations, particle inhomogeneities due to flow transport are small, and thus the differences between the computational approaches may also be small. For example, the intravoxel flow disturbances in MRI scans acquired with short TR values resulted in comparable IVSD profiles using the Eulerian and Lagrangian approaches (Figure 8D). Since the Eulerian approach involves solving the Bloch equation as a partial differential equation with appropriate spatial discretization, the computational cost can be high, depending on the background grid size. In contrast, Lagrangian particles are scattered in each MRI voxel independently from the background velocity field, and the motion of each particle can be computed independently by solving an ordinary differential equation. These properties make the Lagrangian approach efficient in terms of computational cost (Figure 5C) in cases where the simplifications described above can be adopted. Thus, the Lagrangian approach may be suitable for educational use and the design of pulse sequences that require multiple trial and error with limited computational cost.

Note that the output IVSD profiles from the computations showed similar trends to those computed from the input velocity fields, while these values had non‐negligible differences (Figure 8). A possible reason for this misfitting is the assumption of the Gaussian distribution of the flow velocity profile within a voxel (Equation 23). The Gaussian distribution assumption is used for efficiency in numerical formulations and does not perfectly fit the actual velocity profiles. For example, the Gaussian distribution assumption may not be appropriate in cases where the MRI voxel is fine enough that the flow distribution can be considered as a linear velocity gradient. Although an evaluation of the assumption in the IVSD computation is beyond of the scope of this study, computational MRI simulation is potentially useful for the IVSD estimation from prescribed flow velocity profiles and further improvements to the IVSD computation algorithm.

This study had four major limitations and perspectives. First, it aimed to demonstrate the feasibility of the Eulerian approach to computational modeling of PC‐MRI, and thus we made several simplifications. For example, in the case of stenotic flow, we assumed that the magnetization was in the steady state before each repetition of the pulse sequence, which represents an idealized situation.^13^ Because these numerical examples aimed to evaluate the displacement artifacts and intravoxel flow disturbances resulting from each approach, we neglected other sources of errors. Nevertheless, magnetization transport makes the steady state difficult to achieve in practice and may affect the MRI signal intensity and resulting image noise. Also, the process of magnetization excitation by the RF pulse has been simplified, and three‐dimensional rotations of the magnetization by the excitation pulse have not been treated directly. Notably, if the excitation process is sufficiently short so that the effects of magnetization transport during the excitation process can be neglected, the Bloch equation can be treated as an ordinary differential equation using both the Lagrangian and Eulerian approaches and solved using an existing method, such as that described by Reference 4 Further consideration of the above potential errors and hardware‐dependent issues will require comparisons between MRI simulations under idealized conditions and real MRI measurements of the same target. Second, temporal instability of the flow velocity field was neglected to focus instead on the degree of correspondence between spatially complex flow velocity fields and their MRI reconstructions. PC‐MRI simulations with unsteady flow profiles require a large amount of data storage to prepare the prescribed flow velocity fields at every time step during the MRI scan. Nevertheless, MRI of pulsatile flow has several uncertainties associated with temporal flow instabilities and synchronization errors originating from multiple pulsatile cycles such as cardiac and respiratory cycles.^31^ Further investigation of these temporal effects would improve the interpretation of MRI of physiological flow fields. Third, this study focused on simulations of PC‐MRI using an SPGR, whereas there are large variations in pulse sequence and measurement settings that affect resulting MRI output. For example, because the relationship between $T_{C}$ and TE determines the extent of displacement artifacts (Figure 6), exploration of appropriate k‐space sampling strategies based on actual MRI properties and limitations^32^ would be informative. Furthermore, inclusion of diffusion effects in the model^33^ would expand its applicability not only to the PC‐MRI method but also measurement of other flow phenomena, such as intra‐voxel incoherent motion.^34^ Because diffusion effects are expressed as second‐order derivatives of the magnetization in space, extension of the Eulerian approach may be much easier than extension of the Lagrangian approach, which requires a spatial discretization scheme using multiple Lagrangian particles.^35^ Fourth, this study used a Cartesian grid to carry out both the CFD and PC‐MRI simulations. Although this approach can efficiently treat both CFD and MRI simulations using the same computational grid, and a high‐order discretization scheme can easily be applied in a finite difference manner, the method requires relatively large computational resources compared with other CFD methods using unstructured grids, which are usually used to simulate blood flow. Nevertheless, there are several well‐established computational schemes for solving the scalar advection equations on unstructured grids using finite volume^36^ and discontinuous Galerkin^37^ methods, and thus the proposed framework of the PC‐MRI simulation method can be extended using unstructured grids.

## 5 CONCLUSIONS

This study developed an Eulerian formulation of computational PC‐MRI simulation and demonstrated its feasibility and value using three numerical examples. We showed that the developed PC‐MRI simulation method can produce physically consistent outputs, not only for simple straight flow but also stenotic flow with displacement artifacts and intravoxel flow disturbances. These findings demonstrate the feasibility of using the Eulerian formulation of PC‐MRI to investigate the influence of MRI scan parameters on reconstructed flow velocities.

## FUNDING INFORMATION

This work was supported by the Japan Society for the Promotion of Science Grants‐in‐Aid for Scientific Research (No. 23K11830, 24K02408); Ministry of Education, Culture, Sports, Science and Technology as “Program for Promoting Researches on the Supercomputer Fugaku” (Development of human digital twins for cerebral circulation using Fugaku, JPMXP1020230118) and used computational resources of supercomputer Fugaku provided by the RIKEN Center for Computational Science (Project ID: hp230208, hp240220); Nakatani Foundation for Advancement of Measuring Technologies in Biomedical Engineering; and the Multidisciplinary Research Laboratory System for Future Developments, Osaka University Graduate School of Engineering Science.

## CONFLICT OF INTEREST STATEMENT

The authors declare no potential conflicts of interest.
